# Probiotic *Lactobacillus rhamnosus* GKLC1 enhances endurance and metabolism in mice with low exercise capacity

**DOI:** 10.1186/s13104-026-07757-y

**Published:** 2026-03-06

**Authors:** You-Shan Tsai, Yi-Ju Hsu, Chi-Chang Huang, Shih-Wei Lin, Yen-Lien Chen, Chin-Chu Chen

**Affiliations:** 1 Biotech Research Institute, Grape King Bio Ltd., Taoyuan City, Taiwan (R.O.C.); 2 Graduate Institute of Sports Science, National Taiwan Sport University, Taoyuan City, Taiwan (R.O.C.); 3 Institute of Food Science and Technology, National Taiwan University, Taipei City, Taiwan (R.O.C.); 4 Department of Food Science, Nutrition and Nutraceutical Biotechnology, Shih Chien University, Taipei City, Taiwan (R.O.C.); 5 Department of Bioscience Technology, Chung Yuan Christian University, Taoyuan City, Taiwan (R.O.C.)

**Keywords:** Probiotics, Aerobic, Exercise, Lactate, Blood urea nitrogen, Glycogen storage

## Abstract

**Objective:**

Individuals with intrinsically low exercise capacity are at increased risk of metabolic dysfunction and reduced endurance performance. This study investigated whether supplementation with *Lactobacillus rhamnosus* GKLC1 improves exercise performance and energy metabolism in a mouse model selectively bred for instrinsic low aerobic exercise capacity (i-LAEC).

**Results:**

Male i-LAEC ICR mice were orally administered *L. rhamnosus* GKLC1 (0.021 g/kg/day) or sterile water (*n* = 6/group) for six weeks. GKLC1 supplementation significantly prolonged treadmill running time to exhaustion compared with controls. During swimming challenge tests, treated mice exhibited lower serum lactate and blood urea nitrogen levels, indicating improved fatigue recovery. GKLC1 also reduced epididymal fat mass and increased glycogen content in liver and skeletal muscle. No significant differences were observed in body weight, food intake, or serum AST and ALT levels between groups. These findings demonstrate that GKLC1 enhances endurance performance and modulates energy-related metabolism without detectable adverse effects in i-LAEC mice.

## Introduction

Intrinsic aerobic exercise capacity varies widely among individuals and is strongly influenced by genetic factors [1]. Evidence shows that even genetically similar subjects can display markedly different endurance performance [2]. Animal models selectively bred for intrinsic low aerobic exercise capacity (i-LAEC) display compromised endurance, excess fat accumulation, and impaired glucose regulation [3, 4]. Such i-LAEC is closely associated with increased risks of obesity, diabetes, and cardiovascular disease [5]. Approaches to improve exercise performance in these individuals typically include structured training programs or nutritional strategies aimed at enhancing energy metabolism [6, 7]. Among these, probiotics have emerged as a promising candidate due to their systemic metabolic benefits.

Probiotics, defined as live beneficial microorganisms that confer health effects when consumed in adequate amounts, have been widely recognized for their roles in supporting gut health, immune modulation, and systemic metabolism [8]. Recent evidence further suggests that probiotics may enhance exercise performance and mitigate fatigue by influencing energy utilization and recovery-related pathways [9, 10]. For example, supplementation of *Lacticaseibacillus casei* Zhang has been reported to alter host gut microbiota and metabolic profiles, particularly by reducing glucose and glucose-1-phosphate degradation [11]. In addition, a clinical trial demonstrated that daily intake of *Bifidobacterium animalis* sup. *lactis* BL-99 for eight weeks increased maximal oxygen consumption, muscle strength, and plasma levels of lipid-related metabolites such as docosahexaenoic acid, adrenic acid, linoleic acid [12]. However, most existing studies have been conducted in healthy or athletic populations, and little is known about the effects of probiotics in individuals with inherently low exercise capacity. In particular, the mechanisms through which probiotics may influence energy metabolism and exercise endurance in low-capacity models remain largely unexplored.

*Lactobacillus rhamnosus* GKLC1, originally isolated from healthy human breast milk, has previously shown protective effects in gastrointestinal and renal models [13, 14]. Given its capacity to modulate systemic metabolism, we hypothesis that *L. rhamnosus* GKLC1 may also improve exercise endurance, enhance energy utilization, and ameliorate fatigue-related metabolic parameters in subjects with intrinsic low aerobic capacity.

To test this hypothesis, we conducted a study in i-LAEC mice, a model predisposed to poor exercise capacity and metabolic dysfunction [15]. We evaluated the effects of *L. rhamnosus* GKLC1 supplementation on endurance performance, fatigue-related biomarkers, fat accumulation, and glycogen storage, providing an integrated assessment of exercise and metabolic outcomes. This approach allowed us to directly investigate the potential of *L. rhamnosus* GKLC1 as a nutritional intervention for individuals with intrinsic low fitness.

## Materials and methods

### Animals and housing

Male ICR mice selectively bred for intrinsically low aerobic exercise capacity (i-LAEC) were obtained from the National Taiwan Sport University (Taoyuan, Taiwan). The i-LAEC line was established by selective breeding of mice with poor endurance performance through twenty-eight generations of weight-loaded swimming assessments [16]. Eight-week-old mice were housed under conditions at 22 ± 2 °C, 60 ± 5% humidity, and a 12 h light/dark cycle, with standard chow and water available *ad libitum*. All experimental protocols were reviewed and approved by the Institutional Animal Care and Use Committee of National Taiwan Sport University (IACUC No. 11011).

### Probiotic preparation

*L. rhamnosus* GKLC1 was originally isolated from healthy human breast milk. The strain used in the present study was obtained from an established microbial strain collection and no new human samples were collected for this study. Therefore, ethics approval and informed consent were not required. The strain was cultured in de Man Rogosa Sharpe (Merck, Germany) broth at 37 °C for 16 h, followed by scale-up fermentation. Cells were harvested by centrifugation and freeze-dried with 10% skim milk as a cryoprotectant. The resulting powder contained approximately 1.5 × 10¹¹ CFU/g based on plate counts. For administration, GKLC1 powder was freshly suspended in sterile water.

### Experimental design

**Fig. 1:**
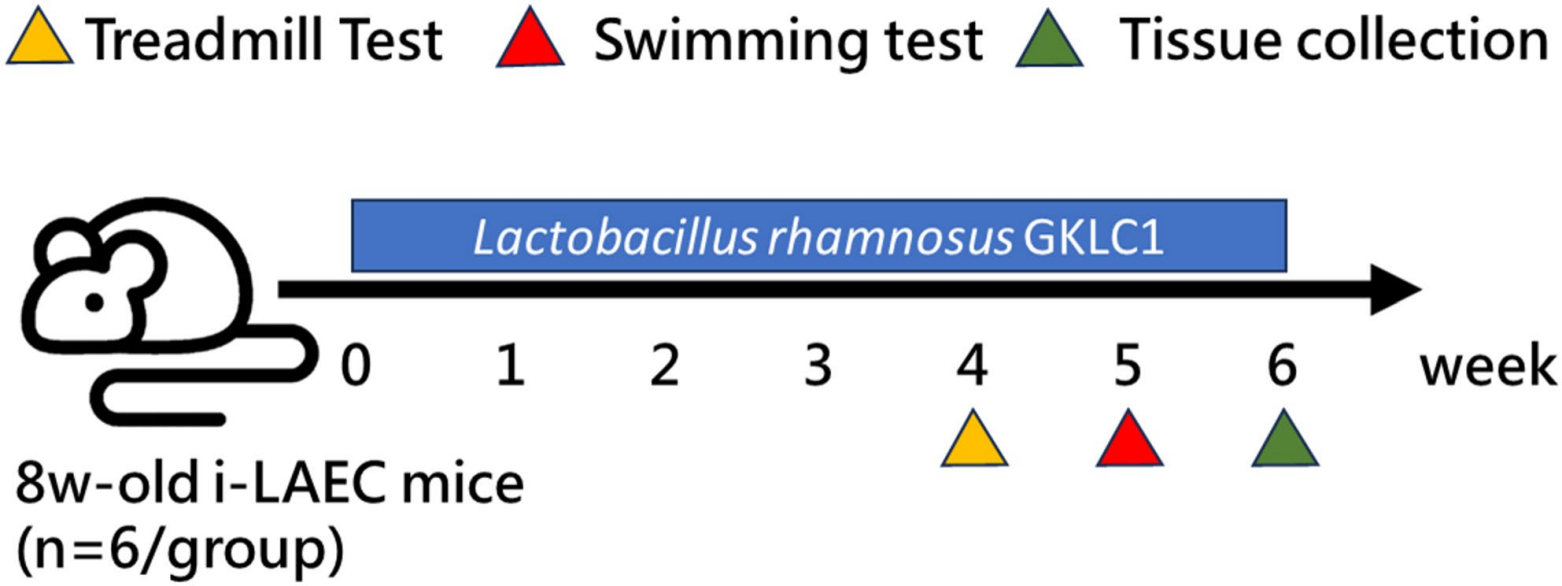
Study design

The i-LAEC mice were assigned into two groups (*n* = 6 each): control (sterile water) and GKLC1 (0.021 g/kg/day, equivalent to approximately 3.15 × 10^9^ CFU/kg/day, by oral gavage). The dosage of *L. rhamnosus* GKLC1 was selected based on previous studies demonstrating physiological benefits in mice at comparable doses [17]. Interventions were conducted daily for six consecutive weeks (Fig. 1). Body weight, food intake, and water consumption were monitored weekly.

Treadmill endurance test was performed at week 4th using a treadmill (15° incline) starting at 12 m/min, with speed increased by 3 m/min every 2 min until exhaustion, defined as remaining in the shock area for > 5 s without resuming running [18]. A low-intensity electrical stimulus (2 Hz, 1.22 mA) was applied only as a gentle encouragement to maintain forward running, in accordance with ethical standards. Swimming tests conducted at week 5. For the long-term exercise test, mice swam for 90 min followed by 60 min rest. Blood samples were taken at baseline and after recovery for blood urea nitrogen (BUN) measurement (Hitachi 7060, Hitachi, Tokyo, Japan). For short-term intensive exercise test, mice with 5% weight-loaded swam individually in 25 cm-deep water (28 cm in diameter) at 27 ± 1 °C for 10 min, followed by 20 min rest. Blood samples were collected at 0, 10, and 30 min to analyze lactate levels (Hitachi 7060, Hitachi, Tokyo, Japan).

At sacrifice (week 6), mice were fasted for 12 h prior to sacrifice. Euthanasia was performed using 95% CO₂ inhalation. Blood samples were collected via cardiac puncture and centrifuged at 15,000 × g for 15 min at 4 °C to obtain serum for biochemical analyses. The epididymal fat pad (EFP), brown adipose tissue (BAT), liver, and muscle tissues were excised and weighted. Liver and muscle tissues were each homogenized in 5 volumes (w/v) of homogenization buffer, centrifuged at 12,000 × g for 15 min at 4 °C, and the resulting supernatant was used for glycogen analysis. Glycogen content was quantified using a standard curve prepared from commercial glycogen standards (Sigma-Aldrich, USA).

### Biochemical assessments

Tissue glycogen content was quantified by perchloric acid extraction and iodine–potassium iodide colorimetric assay with glycogen standards (Sigma-Aldrich, MA, USA). Serum samples were analyzed for safety markers, including aspartate aminotransferase (AST), alanine aminotransferase (ALT), total cholesterol, triglycerides, high-density lipoprotein (HDL), low-density lipoprotein (LDL), and glucose, using commercial enzymatic kits (DiaSys Diagnostic Systems GmbH, Holzheim, Germany).

### Statistics

All values are expressed as mean ± SD (*n* = 6). Differences between groups were analyzed using an unpaired Mann-Whitney test in GraphPad Prism (ver. 10.4.0.). Statistical significance was set at *p* < 0.05.

## Results

### Safety and general observations

All mice remained healthy throughout the six-week intervention. No significant differences in body weight, food intake, or water intake were observed between groups. Serum biochemical markers, including AST, ALT, total cholesterol, triglycerides, HDL, LDL, and glucose, showed no significant alterations, confirming that *L. rhamnosus* GKLC1 supplementation was safe and well tolerated (Table 1).

**Table 1:** Changes in physiological and biochemical parameters in i-LAEC mice during *L. rhamnosus* GKLC1 intervention

	i-LAEC mice	strain GKLC1	*p* value
**Physiological parameters**			
Initial body weight (g)	36.63 ± 4.17	37.58 ± 1.01	0.853
Final body weight (g)	39.78 ± 4.37	39.33 ± 1.35	0.699
Food intake (g/day)	7.90 ± 1.19	7.50 ± 0.68	0.581
Water intake (mL/day)	8.82 ± 1.83	8.44 ± 1.12	0.767
**Biochemical parameters**			
Aminotransferase (U/L)	83.17 ± 3.87	83.80 ± 9.45	0.784
Alanine aminotransferase (U/L)	64.00 ± 4.20	62.83 ± 8.59	> 0.999
Total cholesterol (mg/dL)	156.83 ± 4.22	156.50 ± 7.45	0.911
Triglycerides (mg/dL)	160.67 ± 26.45	176.17 ± 21.95	0.260
High density lipoprotein (mg/dL)	94.33 ± 4.18	89.83 ± 6.21	0.178
Low density lipoprotein (mg/dL)	27.03 ± 3.47	31.40 ± 4.68	0.180
Glucose (mg/dL)	213.50 ± 7.01	218.33 ± 14.68	0.569

Data were expressed as Mean ± S.D. (*n* = 6). Statistical analysis was performed using unpaired Mann-Whitney test. i-LAEC: intrinsic low aerobic exercise capacity

### Exercise endurance and fatigue biomarkers

**Fig. 2:**
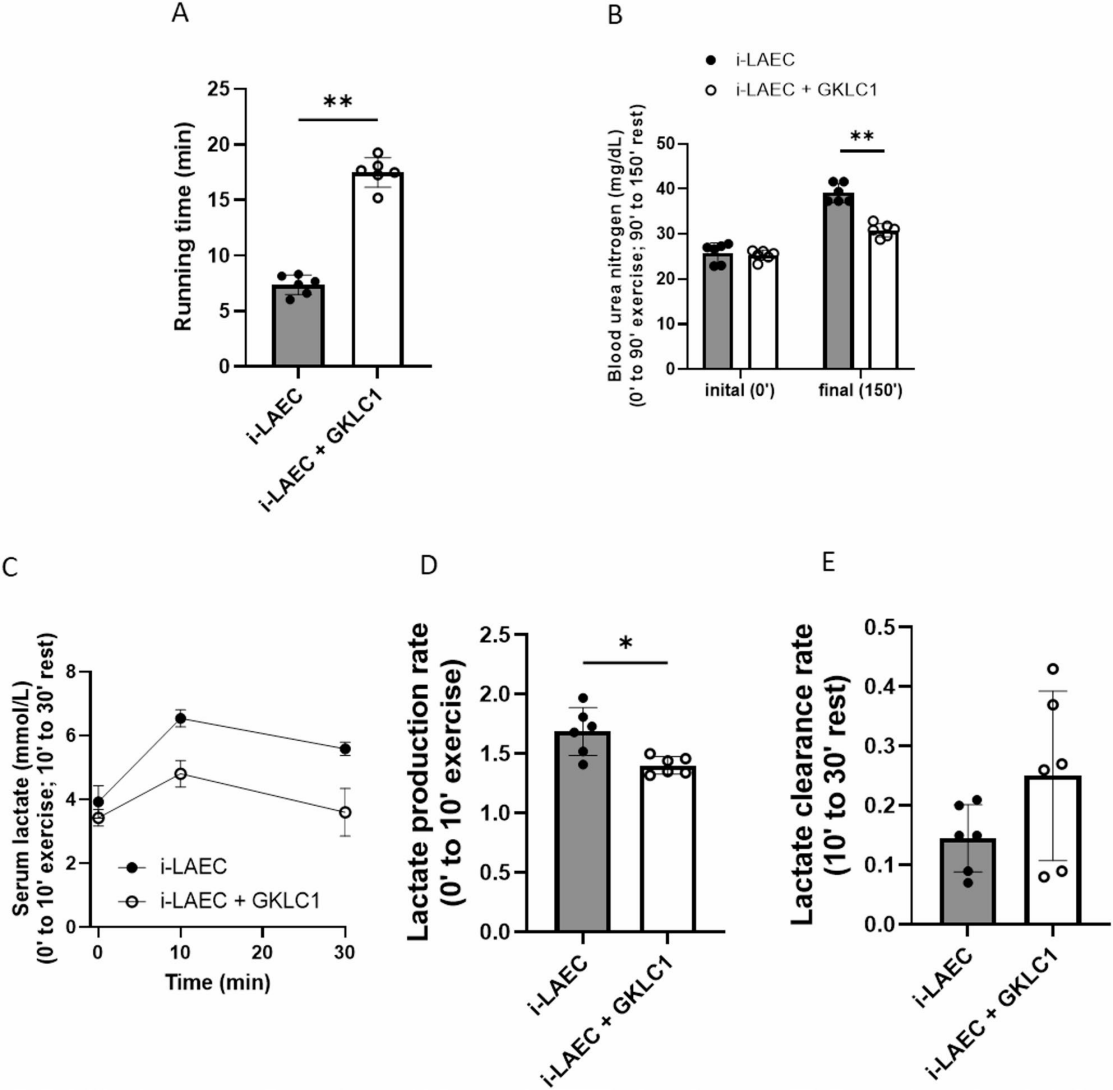
Exercise endurance test in intrinsic low aerobic exercise capacity (i-LAEC) mice. **A** Treadmill running test. **B** Blood urea nitrogen in long-term swimming test. **C** Serum lactate in short-term intensive swimming test. **D** Lactate production rate in short-term intensive swimming test. **E** Lactate clearance rate in short-term intensive swimming test. Data were expressed as Mean ± S.D. (*n* = 6). A significant difference was presented when *p < 0.05 and **p < 0.01 by unpaired Mann-Whitney test

Treadmill exhaustion testing revealed that GKLC1-treated i-LAEC mice had significantly longer running times compared with controls (Fig. 2A). This improvement indicated enhanced aerobic endurance capacity despite the genetically determined low baseline performance of the model.

In the prolonged swimming challenge, BUN levels were markedly elevated in control mice after recovery, while GKLC1-treated mice exhibited significantly lower BUN, suggesting attenuated muscle protein breakdown and improved recovery capacity (Fig. 2B). During the short-term intensive swimming test, serum lactate concentrations were similar between groups at baseline (Fig. 2C). After 10 min of swimming, GKLC1 mice had significantly lower lactate levels compared with controls, reflecting reduced lactate accumulation (Fig. 2D). After 20 min of rest, lactate levels declined more rapidly in the GKLC1 group, although the difference in clearance rate did not reach statistical significance (Fig. 2E).

#### Tissue fat and glycogen storage

**Fig. 3:**
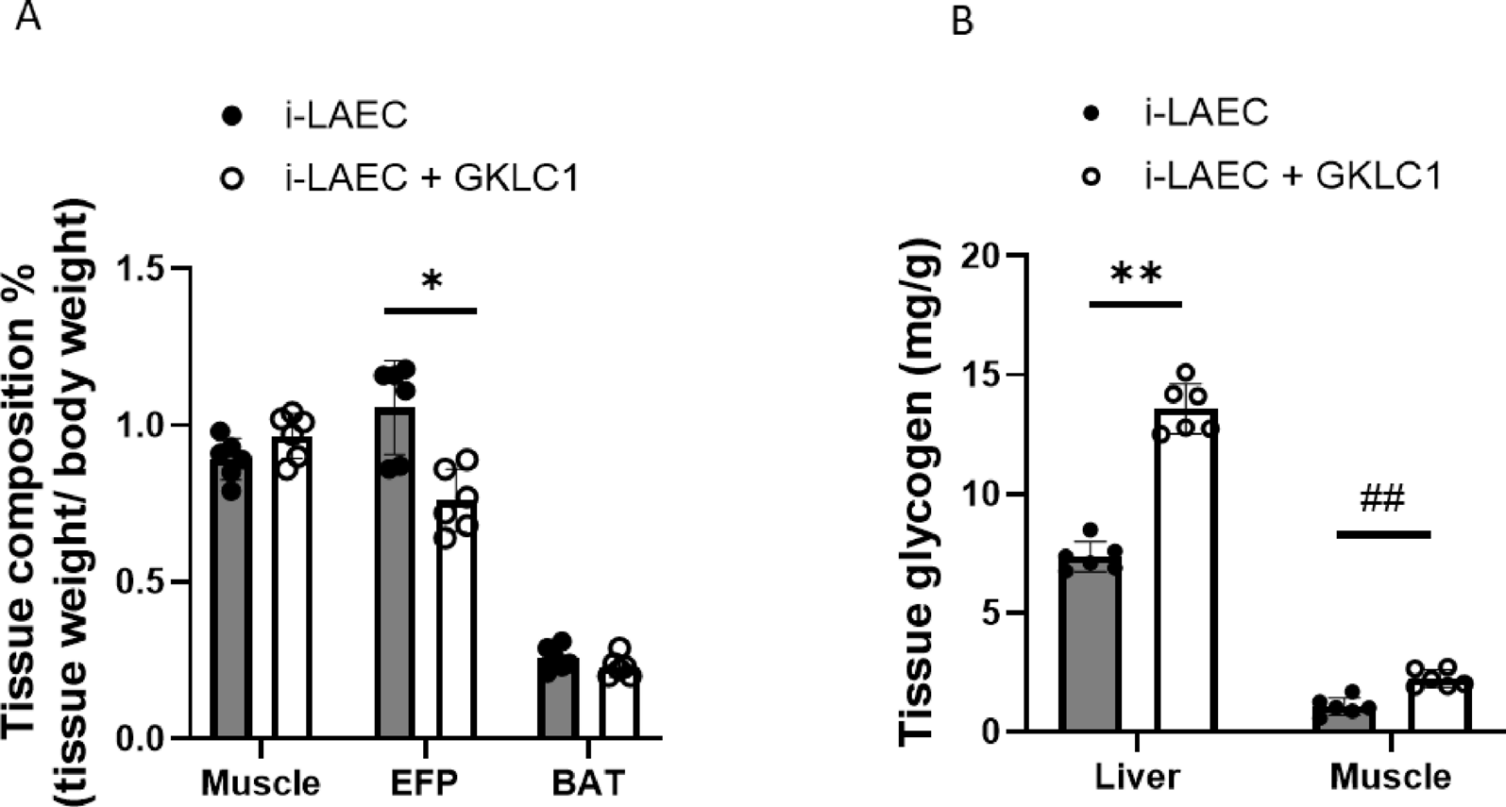
Metabolic outcomes in intrinsic low aerobic exercise capacity (i-LAEC) mice. **A** Tissue composition. Data were expressed as Mean ± S.D. (*n* = 6). Comparisons between i-LAEC and GKLC1 groups were performed for all tissue. Significant differences were observed in EFP (*p < 0.05, Mann–Whitney test). EFP: epidermal fat pad; BAT: brown adipose tissue. **B** Liver and muscle glycogen levels. Data were expressed as Mean ± S.D. (*n* = 6). For liver glycogen, *p < 0.05, **p < 0.01 vs. i-LAEC; for muscle glycogen, #*p* < 0.05, ##*p* < 0.01 vs. i-LAEC (Mann–Whitney test)

Analysis of tissue composition showed that GKLC1 mice had significantly reduced epididymal fat pad mass compared with controls (Fig. 3A). Brown adipose tissue and skeletal muscle weights were not significantly different between groups. Importantly, glycogen levels in both liver and skeletal muscle were significantly higher in the GKLC1 group, suggesting enhanced energy storage and availability for exercise demands (Fig. 3B).

## Discussion

Physical endurance during sports activities can be significantly influenced by an individual’s health and body conditions, which are critical determinants of exercise performance [19]. Nutrition, including probiotics, may influence aspects of host metabolism [20]. In the case of intrinsic low aerobic exercise capacity mice, physical endurance is predictably compromised due to their weaker physiological status [21]. For example, proteins inversely associated with metabolic diseases, such as PGC-1α, PPAR-γ, and COXI, are expressed at lower levels in low-capacity runners [3]. Lei et al. reported that *L. rhamnosus* supplementation in fatigued mice activates the AMPK/PGC-1α signaling pathway, a central regulator of mitochondrial biogenesis, fatty-acid oxidation, and glycogen homeostasis [22]. Activation of this pathway is known to enhance cellular energy efficiency and promote metabolic adaptations associated with improved aerobic capacity.

Our treadmill running tests showed that supplementation with *L. rhamnosus* GKLC1 significantly improved exercise performance (Fig. 2A), providing evidence that *L. rhamnosus* GKLC1 can positively modify the physiological condition of i-LAEC mice. The observed increases in hepatic and muscular glycogen content are related to enhancement of glycogen synthesis, processes known to be regulated by AMPK and PGC-1α signaling. Although the present study did not directly assess these molecular markers, our findings align with the previously reported metabolic effects of *L. rhamnosus* strains. Therefore, it is plausible that GKLC1 improves aerobic capacity by promoting mitochondrial function and increasing baseline energy stores.

Previous studies have highlighted a close relationship between exercise performance and the body’s energy utilization pathways [23, 24]. Blood lactate concentration is a useful indicator for assessing exercise-induced fatigue. Lactate metabolism serves multiple functions, including acting as an energy substrate, gluconeogenic precursor, and signaling molecule [25, 26]. During short-term intensive exercise, muscles rapidly deplete phosphocreatine and glycogen, concomitantly increasing lactate production to generate adenosine triphosphate (ATP) for energy supply [27, 28]. Thus, lactate kinetics, its production and clearance, reflect the efficiency of energy utilization during exercise. Our results showed that i-LAEC mice supplemented with *L. rhamnosus* GKLC1 produced less lactate during intensive exercise (Fig. 2D). Although the increased lactate clearance observed during the rest period did not reach statistical significance (Fig. 2E), the trend suggests that *L. rhamnosus* GKLC1 may enhance energy metabolism in low aerobic capacity subjects.

Blood urea nitrogen, another fatigue-related biomarker, was also assessed. Elevated BUN levels after prolonged exercise reflect increased nitrogen catabolism, primarily from muscle protein breakdown, which enters the bloodstream [29]. Foran et al. reported that exercise-induced BUN elevation requires extended recovery time for normalization [30]. Hence, BUN levels serve as indicators of incomplete recovery. i-LAEC mice treated with *L. rhamnosus* GKLC1 exhibited reduced BUN levels after extended exercise challenges (Fig. 2B). Beyond its role as a fatigue marker, BUN also signals potential kidney injury [31]. Consistent with our previous findings in a cisplatin-induced kidney injury model [14], these results suggest that *L. rhamnosus* GKLC1 may protect against renal stress induced by exercise and facilitate faster fatigue recovery.

During high-intensity exercise, glycogen serves as a crucial energy reserve for efficient ATP production [32, 33]. Depletion of hepatic and muscle glycogen stores has been linked to impaired performance and fatigue onset [34, 35]. *L. rhamnosus* GKLC1 supplementation increased glycogen content in both liver and muscle of i-LAEC mice (Fig. 3B). Since glycogen availability delays fatigue, *L. rhamnosus* GKLC1 appears to improve bioenergetic metabolism to meet muscular energy demands [36]. These effects on glycogen storage may be mediated, at least in part, through modulation of the gut microbiota, which is generally considered a key mechanism by which probiotics influence host metabolism. Gut bacteria can produce short-chain fatty acids (SCFAs) and other bioactive metabolites that act as energy substrates or signaling molecules to enhance glucose uptake and glycogen synthesis in peripheral tissues. Previous research with *Lactobacillus* strains supports the concept that gut microbiota can enhance host glycogen stores and exercise performance via these metabolic interactions. However, it should be noted that gut microbiota composition and SCFA production were not directly assessed in the present study, and these proposed mechanisms are inferred from existing literature.

Finally, i-LAEC subjects commonly exhibit metabolic dysfunction, including excessive white adipose tissue accumulation [16]. Adipose tissue, an endocrine organ, plays a central role in energy metabolism regulation [37]. White adipose tissue (WAT) primarily stores energy as lipid droplets, whereas brown adipose tissue (BAT) is rich in mitochondria and dissipates energy as heat [38]. Some probiotics have been shown to promote fatty acid oxidation by inducing browning of WAT, thereby improving metabolic disorders [39]. Although GKLC1 supplementation reduced epididymal fat pad mass, we did not observe a significant increase in BAT in i-LAEC mice (Fig. 3 A). This suggests that GKLC1 enhances exercise performance in low-capacity subjects primarily through reducing WAT and alleviating metabolic dysfunction rather than promoting BAT activation. Supporting this, our previous study showed that GKLC1 ameliorated liver lipid accumulation and systemic metabolic parameters such as glutathione and catalase in an alcohol-induced liver disease model [13]. Together, these results suggest that GKLC1 may enhance exercise performance and energy utilization in low-capacity subjects by modulating adiposity and systemic metabolism.

### Limitations

The limitation of this study includes the small sample size and the use of a preclinical mouse model, which may not fully reflect human physiology. Only male mice were used, and sex-specific differences in exercise physiology and metabolic responses may limit the generalizability of our findings. Additionally, microbiota composition was not analyzed, and the precise molecular mechanisms underlying the observed effects remain to be clarified. Euthanasia was performed using 95% CO₂ in accordance with the institutional protocol at the time; we acknowledge that more refined, humane methods are recommended for future studies. Future work should include larger cohorts, mechanistic investigations, and translational clinical studies.

## Conclusions

*Lactobacillus rhamnosus* GKLC1 supplementation suggests improved exercise endurance and may exert anti-fatigue effects by reducing the rate of lactate accumulation during exercise and showing a trend toward faster lactate clearance during recovery. The attenuated elevation of BUN following prolonged exercise and rest may indicate physiological adaptation to exercise-induced stress. Additional evidence from lipid reduction and energy metabolism indicates that *L. rhamnosus* GKLC1 may ameliorate genetically driven low exercise capacity, perhaps through systemic metabolic modulation. Future studies should investigate GKLC1’s effects in humans with low fitness and explore its long-term benefits and underlying mechanisms.

## Data Availability

Data is available upon reasonable request from the corresponding author.
